# Comparison of cardiovascular disease risk factors among FiLWHEL (2014–2016), NNS (2013) and KNHANES (2013–2015) women

**DOI:** 10.1186/s12905-023-02218-1

**Published:** 2023-03-30

**Authors:** Sherlyn Mae P. Provido, Grace P. Abris, Heejin Lee, Akinkunmi Paul Okekunle, Glen Melvin Gironella, Mario V. Capanzana, Grace H. Chung, Sangmo Hong, Sung Hoon Yu, Chang Beom Lee, Jung Eun Lee

**Affiliations:** 1 Research Institute of Human Ecology, Seoul National University, 1 Gwanak-ro, Gwanak-gu, Seoul, South Korea; 2 School of Public Health, Loma Linda University, Loma Linda, California USA; 3 Department of Food and Nutrition, College of Human Ecology, Seoul National University, 1 Gwanak-ro, Gwanak-gu, Seoul, 08826 South Korea; 4 Department of Science and Technology-Food and Nutrition Research Institute, DOST Compound, Gen. Santos Avenue, Bicutan, Taguig, Metro Manila Philippines; 5 Department of Child Development and Family Studies, College of Human Ecology, Seoul National University, 1 Gwanak-ro, Gwanak-gu, Seoul, South Korea; 6 Division of Endocrinology and Metabolism, Department of Internal Medicine, Hanyang University Guri Hospital, Hanyang University College of Medicine, 153 Gyeongchun-ro, Guri, South Korea

**Keywords:** Cardiovascular diseases, Dyslipidemia, Filipino women, Hypertension, Immigrant women, Obesity

## Abstract

**Objectives:**

This study assessed the CVD risk factors among Filipino women (FW) in Korea and compared them with FW in the Philippines and women in Korea (KW).

**Methods:**

A cohort of 504 women from the Filipino Women's Health and Diet Study (FiLWHEL) aged 20–57 years old were age-matched (1:1 ratio) with women from the 2013 National Nutrition Survey in the Philippines and the 2013–2015 Korean National Health and Nutrition Examination Survey. Anthropometric data, blood pressure (BP), lipid and glucose levels were compared across the four populations by calculating the odds ratio (OR)s and 95% confidence interval (CI)s using conditional logistic regression models.

**Results:**

Compared to KW, FW in Korea and FW in the Philippines were more than 2 and 3 times higher odds of having obesity for BMI ≥ 30 kg/m^2^ and waist circumference ≥ 88 cm, respectively. However, FW in Korea had the highest odds (OR 5.51, 95% CI 3.18–9.56) of having hypertension compared to KW. FW in the Philippines had the highest odds of having dyslipidemia (compared to KW, total cholesterol ≥ 200 mg/dL: OR 8.83, 95% CI 5.30–14.71; LDL-C ≥ 130 mg/dL: OR 3.25, 95% CI 2.13–4.98; and triglyceride ≥ 150 mg/dL: OR 2.59, 95% CI 1.59–4.22), but FW in Korea and KW had similar prevalence of dyslipidemia.

**Conclusions:**

FW in Korea had higher prevalence of obesity and hypertension, with similar prevalence of dyslipidemia compared to KW in this sample. FW in the Philippines had higher prevalence of dyslipidemia compared to FW in Korea. Further prospective studies are warranted to examine the CVD risk factors among continental and native-born Filipino women.

**Supplementary Information:**

The online version contains supplementary material available at 10.1186/s12905-023-02218-1.

## Background

Cardiovascular diseases (CVD) are primary causes of mortality and morbidity among women worldwide [1, 2]. The global level of all-age CVD-cause disability-adjusted life-years (DALYs) in 2017 was 156 million the highest fraction among non-communicable diseases DALYs for women globally [3]. In 2019, an estimated 523 million CVD cases and 18.6 million CVD-related deaths were reported, with a projected increase to more than 23.6 million deaths by 2030 [4]. Of these deaths, 6.1 million deaths prematurely occurred in people between the ages of 30–70 years due to CVD [5]. Additionally, there were approximately 275 million women with CVD and 8.9 million (47.8%) of all mortality cases among women globally are also attributable to CVD [6].

Elevated blood pressure (BP), dyslipidemia and elevated blood glucose, unhealthy diet, overweight and obesity, physical inactivity, tobacco use, and harmful use of alcohol are well-established modifiable risk factors of CVD [7] but ageing, gender, family history of CVD, ancestry, and socioeconomic status are identified as relevant non-modifiable CVD risk factors [8]. However, the dynamics and burden of these risks factors are often under-reported among migrants populations, particularly in comparison with populations with similar ancestry. For example, the burden and risk factors of CVD among Filipino Women (FW) in Korea compared to native-born Korean Women (KW) is not well understood.

Migrants from different cultural environments often find it hard to align with the cultural and traditional norms of the host country [9]. Specific health disparities and susceptibilities are prevalent in each typology and phase of the migration process [10]. Transition to improved social and economic conditions, and quality health care services has come with complexities associated with changes in dietary habits and nutrition transitions that could substantially modify the CVD risk, incidence, and severity [11].

A study conducted by the National Institutes of Health on CVD risk factors among Asian Americans in the US revealed that migrants have a lower prevalence of CVD risk factors than the host population [12]. However, in this community-based longitudinal study in the US, the migrant population seems to have worse CVD outcomes compared with the host population taking into account gender-specific. For example, Filipino American women presented a greater prevalence of diabetes and metabolic syndrome compared with women of Caucasian ancestry [13]. In addition, Filipino American women were found to have higher rates of heart disease, stroke, coronary heart disease, and hypertension than Hispanic, Non-Hispanic Whites, East Asians, and Asia–Pacific Islanders, and other Asians [14]. CVD susceptibility among migrant FW in the US has been documented in the literature [14]. However, the CVD risk factors or susceptibility to CVD among Filipino and Korean women is not understood clearly yet.

Moreover, whether FW migrants in South Korea are susceptible to higher CVD risk factors than Korean women has not been reported. Understanding the CVD risk susceptibilities among migrant populations deserves attention and is required to design context-specific health promotion guidelines and policies to promote migrant health. In particular, the difference in disease prevalence of migrants in the country of origin and those in the host country may provide insights and perspectives that can help researchers and policymakers to understand the etiology of the disease and design the appropriate strategies.

This study assessed and compared the CVD risk factors among FW migrants in Korea, FW in the Philippines, and KW.

## Methods

### Study population, data sources, and matching

The current analysis included 504 migrant FW in Korea (enrolled in the Filipino Women's Health and Diet Study—FiLWHEL between 2014 and 2016) matched for age (on a ratio of 1:1, age was randomly selected from three datasets and stratified into 20–34, 35–39, 40–44 and 45–57 yielding the same no. of samples for each age group, Table 1. From Tables 3, 4, 5 and 6, the no. of samples varies after the missing data were excluded) with 504 FW in the Philippines (from the Philippine 8th National Nutrition Survey—NNS enrolled in 2013) and 504 KW (from the 6th Korean National Health and Nutrition Examination Survey—KNHANES, enrolled from 2013 to 2015). We randomly selected and matched participants with matching characteristics and information on blood lipids and glucose profiles for statistical analysis in this study. Details of the participants' selection have been reported in Additional file 1: Fig. S1. These three studies used structured questionnaires through on-site face-to-face interviews to collect demographic, socioeconomic, and health-related behaviors. Anthropometry was measured using a non-stretchable tape measure and bioelectric impedance analysis machine. In this analysis, we only use the one-day 24-h recall from three datasets for dietary assessment. It was conducted through an in-person or telephone interview; FiLWHEL, NNS and KNHANES replicate, if not entirely, the USDA five-step multiple-pass methods [15]. BP was measured while the participant was seated calmly using a sphygmomanometer. Details of the protocols, study designs, recruitment methods, data collection procedures and validation of these studies have been described elsewhere [16–18]. All three studies were ethically conducted following with the principles of the Declaration of Helsinki [19].

**Table 1:** Demographic and anthropometric characteristics of FiLWHEL, NNS, and KNHANES women

	FiLWHEL (2014–2016)	NNS (2013)	KNHANES (2013–2015)	*P*-value^a^
No. of participants^d^	504	504	504	
Age, years	34.46 ± 8.10	34.47 ± 8.09	34.01 ± 8.05	matched
Age (years), %				matched
20–34	277 (54.96)	277 (54.96)	277 (54.96)	
35–39	99 (19.64)	99 (19.64)	99 (19.64)	
40–44	68 (13.49)	68 (13.49)	68 (13.49)	
45–57	60 (11.90)	60 (11.90)	60 (11.90)	
Height, cm	153.68 ± 5.38	151.64 ± 5.40	160.52 ± 5.76	< .0001
Weight, kg	55.81 ± 9.82	54.46 ± 10.81	57.16 ± 9.52	< .0001
BMI, kg/m^2^	23.62 ± 3.81	23.63 ± 4.36	22.18 ± 3.47	< .0001
BMI (kg/m^2^), %				< .0001
< 18.5	22 (4.42)	46 (9.18)	57 (11.31)	
18.5– < 23.0	215 (43.17)	206 (41.12)	276 (54.76)	
23.0– < 25.0	107 (21.49)	81 (16.17)	72 (14.29)	
25.0– < 27.0	70 (14.06)	70 (13.97)	50 (9.92)	
27.0– < 30.0	55 (11.04)	59 (11.78)	32 (6.35)	
≥ 30.0	29 (5.82)	39 (7.78)	17 (3.37)	
Waist circumference, cm	79.74 ± 9.45	78.36 ± 11.21	75.16 ± 8.90	< .0001
Waist circumference (cm), %				< .0001
< 80.0	274 (55.35)	276 (58.60)	376 (74.60)	
80.0– < 85.0	91 (18.38)	75 (15.92)	55 (10.91)	
85.0– < 90.0	66 (13.33)	51 (10.83)	36 (7.14)	
≥ 90.0	64 (12.93)	69 (14.65)	37 (7.34)	
Education, %				< .0001
High school or below	164 (32.80)	342 (68.54)	170 (36.17)	
College or above	336 (67.20)	157 (31.46)	300 (63.83)	
Occupation, %				< .0001
Unemployed	261 (52.10)	345 (68.45)	185 (39.53)	
Employed	240 (47.90)	159 (31.55)	283 (60.47)	
Smoking status, %				0.0031
Never smoker	454 (91.90)	417 (87.24)	413 (84.29)	
Past smoker	38 (7.63)	43 (9.00)	50 (10.20)	
Current smoker	6 (1.20)	18 (3.77)	27 (5.51)	
Alcohol intake, %				< .0001
Never drinker	148 (30.52)	278 (58.16)	18 (3.67)	
Past drinker	67 (13.81)	69 (14.44)	72 (14.66)	
Current drinker	270 (55.67)	131 (27.41)	401 (81.67)	

*FiLWHEL* Filipino Women's Diet and Health Study, *NNS* National Nutrition Survey, *KNHANES* Korean National Health and Nutrition Examination Survey, *WHO* World Health Organization, *IASO/IOTF* International Association for the Study of Obesity/International Obesity Task Force, *IDF* International Diabetes Federation, *NCEP* National Cholesterol Education Program, *NHLBI* National Heart, Lung, and Blood Institute

^a^*P*-value for continuous was determined by ANOVA and Mantel-Haenzel test for categorical

^b^BMI (kg/m^2^): Adapted from WHO (1995), WHO (2000) and WHO (2004) guidelines for Asian and Pacific Islander (underweight: < 18.5; normal: 18.5– < 23 and 23– < 25; overweight: 25– < 27; pre-obese: 27– < 30; and obese: ≥ 30)

^c^Waist Circumference categories are absolute cut-offs adapted from various sources published by Lear et.al, 2010 and Yoon et.al, 2014. WHO/IASO/IOTF classification for Asian women (2002), NCEP (2001) and NHLBI (1998) guidelines for women (normal: 80– < 85 cm; abdominal obesity: ≥ 85 cm)

^d^Number of participants did not sum up to 1512 for a few variables because some participants did not provide information on those variable. Frequency age-matching was taken into account

First, we retrieved the NNS data from the Food and Nutrition Research Institute (FNRI) of the Philippines based on mutual agreement, whereas the KNHANES data were freely accessible online via Korea Disease Control and Prevention Agency (KDCA) [17]. Second, men, unmarried women (for the NNS only) and women with missing information on blood lipids and glucose were excluded from the NNS and KNHANES before the matching.

### Demographic and anthropometric measures

Covariates associated with BP, lipids and glucose profiles included in this study were smoking, alcohol drinking, education level, occupation, BMI and waist circumference. Participants' smoking status was categorized into ever smoker and never smoker; ever smoker was defined as past or current smoker while never smoker was defined as never smoked at least 100 cigarettes in their entire lifetime. Alcohol drinkers were categorized into past or current drinker (including occasionally or had at least one to twelve alcoholic beverages of any type in their entire lifetime) or never drinkers (lifelong abstainers or who never consumed one or more drinks of any type of alcohol in their entire lifetime). Participants' employment status was categorized as employed and unemployed (including housewife, student, retired, etc.). In addition, participants were asked about their educational attainment where elementary or middle or high school level were grouped into high school and below, and those who had at least college education were grouped into college and above.

### Collection of blood samples and determination of glucose and lipid profiles

For the FiLWHEL, the Cobas 8000 C702-1/C703-I analyzer (Roche Diagnostics, Basel, Switzerland) was used to determine serum levels of TC, TG, and HDL-C by enzymatic method and glucose by hexokinase UV assay. For the NNS, the TC, TG, HDL-C and glucose were assessed by enzymatic colorimetric method using the Roche COBAS Integra and Hitachi 912 analyzer. For the KNHANES, the TC and TG were analyzed by enzymatic method, HDL-C by homogeneous enzymatic colorimetric method, and glucose by hexokinase UV assay on Hitachi Automatic Analyzer 7600/7600-210 (Hitachi Co., Tokyo, Japan). All blood samples were collected by phlebotomists or trained medical personnel after at least 8–12-h overnight fasting through a venipuncture procedure.

The FiLWHEL coefficients of variation (CVs) were as follows: TC (1.43–2.24%), TG (1.85–2.10%), HDL-C (1.55–2.51%), and glucose (1.15–1.22%) [16]. The blood samples for NNS participants were stored on ice and later centrifuged to separate plasma, which was later packed, labeled and frozen at − 20 °C until ready for analysis in the laboratory. The CVs for the NNS were directly derived and calculated from the 8th NNS clinical data and were as follows: TC (2.85–4.27%), HDL-C (1.44–5.08%), LDL-C (2.87–4.07%), TG (1.65–23.55%), and glucose (0.73–10.39%) [18]. The KNHANES blood samples were processed and immediately refrigerated then transported in cold storage to the Central Testing Institute on the same day of the collection. The serum was separated immediately by centrifugation for analysis. The KNHANES CVs were as follows: TC (0.9–1.70%), HDL-C (0.9–2.06%), LDL-C (1.10–2.60%), TG (0.9–2.77%), and glucose (0.34–2.07%) [20]. LDL-C was not directly assayed in the FiLWHEL study. The LDL-C was calculated from the measured values of the TC, TG and HDL-C using the Friedewald equation [LDL-C = (TC)-(HDL-C)-(TG)/5], where all values are expressed in mg/dL [21].

### Criteria for the definition of CVD risk factors

### Obesity

BMI was calculated as weight (kg) divided by square of height (m^2^), and respondents were classified according to the World Health Organization cut-off points: underweight, < 18.5 kg/m^2^; normal, 18.5– < 23.0 (for Asian) and 23.0– < 25.0 kg/m^2^; overweight, 25.0– < 27.0 kg/m^2^; pre-obese, 27.0– < 30.0 kg/m^2^ and obese, ≥ 30.0 kg/m^2^ [22]. Also, for waist circumference, we defined abdominal obesity using the absolute cut-off points from the 2001 NCEP (National Cholesterol Education Program) and 1998 NHLBI Obesity Education Initiative Expert Panel for Asian and American women (normal: 80– < 88 cm; abdominal obesity: ≥ 88 cm) classifying high waist circumference for each population in this study [23].

### Hypertension

Using the 2017 American College of Cardiology/American Heart Association (ACC/AHA) guidelines, systolic and diastolic blood pressures were classified as follows: normal, < 120/ < 80 mmHg; prehypertension, ≥ 120/ ≥ 80 mmHg or < 140/ < 90 mmHg; and hypertension, ≥ 120/ ≥ 90 mmHg [24].

### Dyslipidemia and elevated blood glucose

The 2005 American Heart Association/National Heart, Lung, and Blood Institute (AHA/NHLBI) criteria was used to categorize the blood lipids and glucose levels to determine dyslipidemia: high TC ≥ 200 mg/dL; low HDL-C < 50 mg/dL; high LDL-C ≥ 130 mg/dL; high TG ≥ 150 mg/dL, and elevated glucose ≥ 100 mg/dL [25].

### Statistical analysis

Continuous variables were presented as means ± SD and compared across the three populations (FiLWHEL, NNS, and KNHANES) using the analysis of variance (ANOVA), but categorical data were presented as n (%) and compared using Mantel-Haenzel test. Odds ratios (ORs) and 95% confidence intervals (CIs) were calculated using conditional multivariate logistic regression models with the women from the KNHANES as the reference population. Women with missing BMI, waist circumference, BP, lipid and glucose data were excluded when we analyzed these variables as outcomes in the logistic regression models. The multivariate analyses were adjusted for energy intake (kcal/day, continuous), smoking status (ever, never), alcohol intake (never, past, current), educational level (high school or below, college or above), employment status (employed, unemployed), BMI (< 18.5, 18.5– < 25.0, 25.0– < 30.0, ≥ 30.0 kg/m^2^) and waist circumference (< 85 cm, ≥ 85 cm) as applicable. Tukey's posthoc test was used for multiple comparison analyses to assess significant differences in nutrient intake across the population. All statistical analyses were performed using the SAS version 9.4 software package (SAS Institute Inc., Cary, NC, USA), and two-tailed *P* values < 0.05 were statistically significant.

## Results

### Demographic and anthropometric profiles of the FiLWHEL, NNS, and KNHANES

Table 1 presents the demographic and anthropometric characteristics of the FiLWHEL, NNS, and KNHANES. FW in Korea and the Philippines showed a higher proportion of being overweight or obese (BMI ≥ 25.0kg/m^2^, 30.9% and 33.5%, respectively) and had a higher waist circumference (≥ 85cm, 26.3% and 25.5%) compared to KW, (*P* < 0.0001). Additionally, 32.8% of FW in Korea had low educational attainment (high school or below) compared to 36.2% and 68.5% of the KW and FW in the Philippines, respectively (*P* < 0.0001). In contrast, unemployment was significantly (*P* < 0.0001) prevalent among FW in Korea (52.1%) compared to KW (39.5%). The prevalence of current smokers was significantly lower (*P* < 0.0001) among FW in Korea (1.2%) compared to FW in the Philippines (3.8%) and KW (5.5%). Similarly, the prevalence of current alcohol drinking was significantly lower (*P* < 0.0001) among FW in Korea (55.7%) compared to KW (81.7%).

### Blood pressure, lipids, lipid ratio and glucose of the FiLWHEL, NNS, and KNHANES

Table 2 presents the details of the metabolic profiles of the women in the FiLWHEL, NNS, and KHANES. The blood pressure of FW compared to KW was significantly higher while FW in Korea had significantly higher mean SBP levels (116.29 ± 16.06 mmHg) compared to the FW in the Philippines (111.68 ± 15.65 mmHg) and KW (107.06 ± 12.10 mmHg). The DBP levels showed a similar pattern. Additionally, FW in Korea (8.5%) had a significantly higher prevalence of elevated BP (SBP ≥ 140 or DBP ≥ 90) than KW (1.8%).

**Table 2:** Blood pressures^b^, lipids^c^, glucose^d^ and lipid ratio characteristics among FiLWHEL, NNS, and KNHANES women

	FiLWHEL (2014–2016)	NNS (2013)	KNHANES (2013–2015)
No. of participant^f^	504	504	504
*Blood pressure*			
SBP, mmHg	117.62 ± 17.26	111.64 ± 15.62	107.32 ± 12.29
DBP, mmHg	75.83 ± 10.79	72.72 ± 10.79	71.23 ± 9.19
Blood Pressure, %			
SBP < 120 and DBP < 80	267 (56.21)	316 (62.95)	413 (82.44)
SBP ≥ 120– < 140 or DBP ≥ 80– < 90	161 (33.89)	138 (27.49)	78 (15.57)
SBP ≥ 140 or DBP ≥ 90	47 (9.89)	48 (9.56)	10 (2.00)
SBP, mmHg^e^	116.29 ± 16.06	111.68 ± 15.65	107.06 ± 12.10
DBP, mmHg^e^	75.08 ± 10.38	72.75 ± 10.82	71.04 ± 9.07
Blood pressure,^e^ %			
SBP < 120 and DBP < 80	263 (58.57)	313 (62.73)	410 (83.00)
SBP ≥ 120– < 140 or DBP ≥ 80– < 90	148 (32.96)	138 (27.66)	75 (15.18)
SBP ≥ 140 or DBP ≥ 90	38 (8.46)	48 (9.62)	9 (1.82)
*Lipids and glucose*			
TC, mg/dL	180.32 ± 36.10	192.09 ± 41.54	184.40 ± 32.63
TC, %			
< 200	358 (73.36)	312 (61.90)	364 (72.22)
≥ 200	130 (26.64)	192 (38.10)	140 (27.78)
TC, mg/dL^e^	180.13 ± 35.91	192.18 ± 41.53	184.49 ± 32.70
TC,^e^ %			
< 200	354 (73.60)	311 (61.83)	358 (72.03)
≥ 200	127 (26.40)	192 (38.17)	139 (27.97)
LDL-C, mg/dL	103.89 ± 31.45	126.26 ± 38.07	108.25 ± 29.11
LDL-C, %			
< 130	400 (81.97)	289 (57.34)	402 (79.76)
≥ 130	88 (18.03)	215 (42.66)	102 (20.24)
LDL-C, mg/dL^e^	103.79 ± 31.33	126.38 ± 38.02	108.41 ± 29.19
LDL-C,^e^ %			
< 130	394 (81.91)	288 (57.26)	395 (79.48)
≥ 130	87 (18.09)	215 (42.74)	102 (20.52)
TG, mg/dL	91.21 ± 52.26	127.52 ± 77.47	95.02 ± 67.77
TG, %			
< 150	432 (88.52)	381 (75.60)	442 (87.70)
≥ 150	56 (11.48)	123 (24.40)	62 (12.30)
TG, mg/dL^e^	91.07 ± 52.48	127.61 ± 77.52	94.51 ± 67.67
TG,^e^ %			
< 150	426 (88.57)	380 (75.55)	438 (88.13)
≥ 150	55 (11.43)	123 (24.45)	59 (11.87)
HDL-C, mg/dL	58.19 ± 14.06	40.26 ± 11.80	57.49 ± 12.26
HDL-C, %			
≥ 50	352 (72.13)	97 (19.25)	362 (71.83)
< 50	136 (27.87)	407 (80.75)	142 (28.17)
Glucose, mg/dL	87.81 ± 11.78	88.66 ± 30.30	91.96 ± 15.04
Glucose, %			
< 100	442 (90.57)	433 (85.91)	435 (86.31)
≥ 100	46 (9.43)	71 (14.09)	69 (13.69)
Glucose, mg/dL^e^	87.42 ± 10.93	88.65 ± 30.36	91.35 ± 12.73
Glucose,^e^ %			
< 100	438 (91.44)	431 (85.86)	435 (87.17)
≥ 100	41 (8.56)	71 (14.14)	64 (12.83)
*Lipids ratio*			
TC/HDL-C, mg/dL	3.25 ± 0.96	5.29 ± 2.49	3.35 ± 0.93
LDL-C/HDL-C, mg/dL	1.90 ± 0.77	3.52 ± 1.88	2.00 ± 0.78
TC/TG, mg/dL	2.41 ± 1.01	1.82 ± 0.73	2.52 ± 1.20
TG/HDL-C, mg/dL	1.77 ± 1.49	3.84 ± 4.34	1.85 ± 1.70
TG/LDL-C, mg/dL	0.93 ± 0.62	1.07 ± 0.72	0.90 ± 0.60

*FiLWHEL* Filipino Women's Diet and Health Study, *NNS* National Nutrition Survey, *KNHANES* Korean National Health and Nutrition Examination Survey, *SBP* systolic blood pressure, *DBP* diastolic blood pressure, *LDL-C* low density lipoprotein, *HDL-C* high density lipoprotein, *TC* total cholesterol, *TG* triglyceride

^a^The values are presented as mean ± SD (Standard deviation) for continuous variables and proportions (percentage, %) for categorical

^b^Blood pressure criteria: 2017 ACC/AHA guidelines (SBP/DBP < 120/ < 80 mmHg defined as normal, SBP/DBP ≥ 120/ ≥ 80 mmHg or < 140/ < 90 mmHg defined as prehypertension and SBP/DBP ≥ 140/ ≥ 90 mmHg defined as hypertension)

^c^Blood lipid criteria: 2005 AHA/NHLB criteria/2019 ACC/AHA Guidelines (TC cut-off: < 200 mg/dL as desirable level and ≥ 200 mg/dL is borderline high and high level); HDL-C cut-off for women (< 50 mg/dL as low level and ≥ 50 mg/dL as normal level); LDL-C cut-off (< 130 mg/dL defined as optimal and ≥ 130 mg/dL defined as above optimal to very high level); TG cut-off (< 150 mg/dL defined as optimal and ≥ 150 mg/dL defined as above optimal to very high level)

^d^Blood glucose criteria: 2019 American Diabetes Association Guidelines (glucose level at < 100 mg/dL is defined as normal and ≥ 100 mg/dL defined as elevated)

^e^Participants were excluded in the analysis for medication use and/or treatment for hypertension or dyslipidemia or diabetes mellitus

^f^Number of participants did not sum up to 1512 for a few variables because some participants did not provide information on those variable. Matching by age frequency was also taken into account

The prevalence of elevated TC was significantly higher among FW in the Philippines at 38.2%, whereas, there was seen similarity between KW at 28.0% and FW in Korea at 26.4%. Similarly, the mean TC levels for FW in the Philippines were 192.18 ± 41.53 mg/dL, 180.13 ± 35.91 mg/dL among FW in Korea and 184.49 ± 32.70 mg/dL among KW. Similar trends were observed for the means of LDL-C and TG, with the highest mean levels among FW in the Philippines. However, KW were observed to have slightly higher mean glucose levels compared to FW.

### Nutrient intake profiles of the FiLWHEL, NNS, and KNHANES

FW in the Philippines had the lowest intakes for total energy, protein and fat percent energy, calcium, vitamins A and C, iron, thiamin, and riboflavin but the highest in carbohydrate percent energy intake. FW in the Philippines had lower intakes of protein, fat, and micronutrients compared with FW in Korea and KW. When we compared FW in Korea with KW, we found no significant difference in nutrient intakes except for the protein percent energy, calcium, iron, thiamin and riboflavin (Table 3).

**Table 3:** Nutrient intake characteristics of FiLWHEL, NNS, and KNHANES women

	FiLWHEL^a^ (2014–2016)	NNS^b^ (2013)	KNHANES^c^ (2013–2015)	*P* value^1^	Post-hoc^2^
No. of participants^3^	219	219	219		
Energy (kcal/d)	1766.97 ± 727.68	1561.76 ± 584.58	1901.00 ± 824.32	< .0001	a-b, b-c
Carbohydrate (g/d)	255.08 ± 111.37	279.86 ± 109.37	273.00 ± 117.45	0.0604	
Protein (g/d)	69.74 ± 35.13	48.30 ± 18.23	68.25 ± 34.22	< .0001	a-b, b-c
Total fat (g/d)	50.98 ± 30.06	27.66 ± 23.60	52.05 ± 34.98	< .0001	a-b, b-c
Carbohydrate (% energy)	58.55 ± 11.32	72.24 ± 10.12	58.91 ± 12.29	< .0001	a-b, b-c
Protein (% energy)	15.67 ± 4.35	12.60 ± 2.79	14.31 ± 3.54	< .0001	a-b, a-c, b-c
Total fat (% energy)	25.35 ± 8.84	15.15 ± 9.50	23.74 ± 8.70	< .0001	a-b, b-c
Calcium (mg/d)	381.66 ± 291.82	310.03 ± 176.75	473.56 ± 272.14	< .0001	a-b, a-c, b-c
Vitamin C (mg/d)	85.32 ± 78.26	38.98 ± 49.36	92.67 ± 138.69	< .0001	a-b, b-c
Vitamin A (mcg RE/d)	564.79 ± 568.96	397.06 ± 622.94	668.95 ± 762.85	< .0001	a-b, b-c
Iron (mg/d)	12.19 ± 7.37	8.16 ± 3.86	15.07 ± 8.17	< .0001	a-b, a-c, b-c
Thiamin (mg/d)	1.21 ± 0.76	0.74 ± 0.41	1.92 ± 1.05	< .0001	a-b, a-c, b-c
Riboflavin (mg/d)	1.00 ± 0.58	0.64 ± 0.36	1.40 ± 0.79	< .0001	a-b, a-c, b-c
Niacin (mg/d)	16.19 ± 8.97	15.78 ± 6.18	15.77 ± 8.58	0.8250	

*FiLWHEL* Filipino Women's Diet and Health Study, *NNS* National Nutrition Survey, *KNHANES* Korean National Health and Nutrition Examination Survey

^1^*P-*value was determined using ANOVA: mean ± SD (Standard deviation)

^2^Multiple-comparison analyses using Tukey’s post-hoc tests assess significant differences in nutrient intakes between FiLWHEL^a^, NNS^b^, and KNHANES^c^ women at *p* < 0.05 level

^3^We only included 219 participants with dietary data and frequency age-matching was taken into account

^a–b^FiLWHEL women nutrient intakes were significantly different from that of NNS women

^a–c^FiLWHEL women nutrient intakes were significantly different from that of KNHANES women

^b–c^NNS women nutrient intakes were significantly different from that of KNHANES women

### Odds ratio and 95% confidence interval for obesity, elevated BP, dyslipidemia and elevated glucose

We observed the higher prevalence of obesity among FW in KW and in the Philippines (Table 4). ORs for BMI (≥ 30.0 kg/m^2^) were 2.27 (95% CI 1.15–4.51) for FW in Korea and 3.49 (95% CI 1.59–7.64) for FW in the Philippines; the ORs for high waist circumference (≥ 88 cm) among FW in Korea and in the Philippines were 2.36 (95% CI 1.53–3.63) and 3.34 (95% CI 1.96–5.68), respectively, when compared with KW.

**Table 4:** Multivariate-adjusted odds ratio and 95% confidence interval of obesity among FiLWHEL, NNS, and KNHANES women

	KNHANES (2013–2015)	FiLWHEL (2014–2016)	NNS (2013)
No. of cases/total n	17/495	29/495	39/495
BMI, ≥ 30 kg/m^a^			
Age-adjusted OR	1.00	1.76 (0.95–3.26)	2.44 (11.35–4.40)
MV-adjusted OR^c^	1.00	2.27 (1.15–4.51)	3.49 (1.59–7.64)
No. of cases/total n	46/462	82/462	91/462
Waist circumference, ≥ 88cm^b^			
Age-adjusted OR	1.00	1.98 (1.34–2.92)	2.25 (1.53–3.32)
MV-adjusted OR^c^	1.00	2.36 (1.53–3.63)	3.34 (1.96–5.68)

*FiLWHEL* Filipino Women's Diet and Health Study, *NNS* National Nutrition Survey, *KNHANES* Korean National Health and Nutrition Examination Survey

^a^BMI (kg/m^2^): Adapted from WHO (1995), WHO (2000) and WHO (2004) guidelines (underweight: < 18.5; normal: 18.5– < 23 and 23– < 25; overweight: 25– < 27; pre-obese: 27– < 30; and obese: ≥ 30)

^b^Waist circumference categories are absolute cut-offs adapted from various sources published by Lear et.al, 2010 and Yoo et.al, 2015, (normal: 80– < 88 cm; abdominal obesity: ≥ 88 cm)

^c^Multivariate model (MV)—adjusted for energy (kcal/day, continuous), smoking status (ever, never), alcohol intake (never, past and current), education level (high school or below, college or above), and employment status (employed, unemployed)

The FW in Korea and Philippines had 5.51 (95% CI 3.18–9.56) and 2.38 (95% CI 1.19–4.79) higher odds of having hypertension, respectively, when compared to KW (Table 5). When we further adjusted for obesity (BMI kg/m^2^), the association became attenuated among FW in the Philippines and in Korea.

**Table 5:** Multivariate-adjusted odds ratio and 95% confidence interval of hypertension among FiLWHEL, NNS, and KNHANES women

	KNHANES (2013–2015)	FiLWHEL (2014–2016)	NNS^a^ (2013)
No. of cases/Total n	23/470	93/470	59/470
Age-adjusted OR	1.00	5.65 (3.39–9.41)	3.02 (1.79–5.10)
Model 1	1.00	5.51 (3.18–9.56)	2.38 (1.19–4.79)
Model 2	1.00	4.85 (2.74–8.60)	1.98 (0.96–4.08)
Model 3	1.00	4.85 (2.77–8.48)	2.17 (1.07–4.40)

*FiLWHEL* Filipino Women's Diet and Health Study, *NNS* National Nutrition Survey, *KNHANES* Korean National Health and Nutrition Examination Survey

Blood pressure criteria: 2017 ACC/AHA guidelines (SBP/DBP < 120/ < 80 mmHg defined as normal, SBP/DBP ≥ 120/ ≥ 80 mmHg or < 140/ < 90 mmHg defined as prehypertension and SBP/DBP ≥ 140/ ≥ 90 mmHg defined as hypertension)

^a^FiLWHEL and KNHANES have medication use and treatment data for high blood pressure; NNS has only antihypertensive use

Model 1: Adjusted for multivariate—energy (kcal/day, continuous), smoking status (ever, never), alcohol intake (never, past and current), education level (high school or below, college or above), and employment status (employed, unemployed)

Model 2: Model 1 multivariate + body mass index (< 18.5, 18.5– < 25.0, 25.0– < 30.0 and ≥ 30.0 kg/m^2^)

Model 3: Model 1 multivariate + waist circumference (< 85, ≥ 85 cm)

FW in the Philippines had unfavorable lipid profiles when compared to KW including a higher prevalence of high TC ≥ 200 mg/dL (OR 8.83, 95% CI 5.30–14.71), low HDL-C < 50 mg/dL (OR 7.46, 95% CI 4.87–11.44), high LDL-C ≥ 130 mg/dL (OR 3.25, 95% CI 2.13–4.98) and high TG ≥ 150 mg/dL (OR 2.59, 95% CI 1.59–4.22). However, FW in Korea showed similar lipid profiles to those of KW (Table 6).

**Table 6:** Multivariate-adjusted odds ratio and 95% confidence interval dyslipidemia^b^ and diabetes mellitus^c^ among FiLWHEL, NNS^a^, and KNHANES women

	KNHANES (2013–2015)	FiLWHEL (2014–2016)	NNS (2013)
No. of cases/total n	254/488	253/488	433/488
High TC, ≥ 200 mg/dL			
Age-adjusted OR	1.00	0.99 (0.77–1.28)	7.78 (5.41–11.19)
Model 1	1.00	1.02 (0.77–1.35)	8.83 (5.30–14.71)
Model 2	1.00	0.81 (0.60–1.10)	7.34 (4.30–12.54)
Model 3	1.00	0.84 (0.62–1.13)	7.33 (4.35–12.36)
No. of cases/total n	136/488	136/488	392/488
Low HDL-C, < 50 mg/dL			
Age-adjusted OR	1.00	1.00 (0.75–1.32)	9.37 (6.77–12.97)
Model 1	1.00	0.93 (0.69–1.26)	7.46 (4.87–11.44)
Model 2	1.00	0.76 (0.55–1.05)	6.65 (4.26–10.36)
Model 3	1.00	0.78 (0.57–1.07)	6.67 (4.33–10.29)
No. of cases/total n	106/488	107/488	209/488
High LDL-C, ≥ 130 mg/dL			
Age-adjusted OR	1.00	1.01 (0.74–1.38)	2.83 (2.11–3.79)
Model 1	1.00	0.95 (0.68–1.34)	3.25 (2.13–4.98)
Model 2	1.00	0.84 (0.59–1.19)	3.00 (1.94–4.65)
Model 3	1.00	0.86 (0.61–1.22)	3.12 (2.03–4.79)
No. of cases/total n	67/488	79/488	122/488
High TG, ≥ 150 mg/dL			
Age-adjusted OR	1.00	1.22 (0.85–1.74)	2.18 (1.55–3.06)
Model 1	1.00	1.32 (0.90–1.94)	2.59 (1.59–4.22)
Model 2	1.00	1.05 (0.69–1.59)	2.11 (1.24–3.59)
Model 3	1.00	1.09 (0.73–1.64)	2.33 (1.40–3.87)
No. of cases/total n	67/488	53/488	68/488
High glucose, ≥ 100 mg/dL			
Age-adjusted OR	1.00	0.76 (0.51–1.12)	1.02 (0.70–1.48)
Model 1	1.00	0.77 (0.49–1.19)	0.99 (0.58–1.68)
Model 2	1.00	0.63 (0.39–1.01)	0.73 (0.41–1.31)
Model 3	1.00	0.63 (0.40–1.01)	0.84 (0.48–1.47)

*FiLWHEL* Filipino Women's Diet and Health Study, *NNS* National Nutrition Survey, *KNHANES* Korean National Health and Nutrition Examination Survey, *TC* total cholesterol, *HDL-C* high-density lipoprotein, *LDL-C* low density lipoprotein, *TG* triglyceride

^a^FiLWHEL and KNHANES have medication use and treatment for high cholesterol or dyslipidemia; NNS has only medication use data for high cholesterol or dyslipidemia

^b^Blood lipid criteria: 2005 AHA/NHLB criteria/2019 ACC/AHA Guidelines (TC cut-off: < 200 mg/dL as desirable level and ≥ 200 mg/dL is borderline high and high level); HDL-C cut-off for women (< 50 mg/dL as low level and ≥ 50 mg/dL as normal level); LDL-C cut-off (< 130 mg/dL defined as optimal and ≥ 130 mg/dL defined as above optimal to very high level); TG cut-off (< 150 mg/dL defined as optimal and ≥ 150 mg/dL defined as above optimal to very high level)

^c^Blood glucose criteria: 2019 American Diabetes Association Guidelines (glucose level at < 100 mg/dL is defined as normal and ≥ 100 mg/dL defined as elevated)

Model 1: Adjusted for multivariate—energy (kcal/day, continuous), smoking status (ever, never), alcohol intake (never, past and current), education level (high school or below, college or above), and employment status (employed, unemployed)

Model 2: Model 1 multivariate + body mass index (< 18.5, 18.5– < 25.0, 25.0– < 30.0 and ≥ 30.0 kg/m^2^)

Model 3: Model 1 multivariate + waist circumference (< 85, ≥ 85 cm)

## Discussions

In this study, we compared CVD risk factors among FW in Korea and in the Philippines and KW. We found that FW in the Philippines had a higher prevalence of obesity and unfavorable lipid profiles than FW in Korea, suggesting that migration to Korea led to lower vulnerability to poor CVD health outcomes. However, FW in Korea had a higher prevalence of obesity and hypertension than KW.

The etiology of CVD burden among FW in the Philippines is complex and warrants further studies, but can be explained in the following ways. The average diet of a Filipino is characterized by less consumption of fruits and vegetables and higher consumption of meat, fast foods, fried foods and sugar-sweetened beverages [26] along with the adoption of a Westernized diet [27], responsible for the steady increase of obesity among Filipino in the Philippines for the past two decades from 20.2% in 1998 to 26.6% in 2019 [18]. In this study, FW in the Philippines were found to have the highest prevalence of obesity across the population. Moreover, we found the highest intake of carbohydrates in FW in the Philippines among the three populations, which may be related to a low HDL-C. Genetic factors may be potential reasons for these differences. The Cebu Longitudinal Health and Nutrition Survey (CLHNS) showed genome-wide significant associations of several genetic variants with obesity [28] and triglycerides and cholesterol [29]. However, it remains unclear whether the genetic traits of Filipinos are key relevant factors for obesity, hypertension or dyslipidemia. Interestingly, when we compared FW in Korea with KW, we found a similarity in the lipid profiles, suggesting that changes in dietary habits and health practices [30], an increase in food diversity [31], and an improvement in socioeconomic status [32] may be associated with a lower prevalence of dyslipidemia.

Several epidemiologic studies have reported the associations between dietary habits and lipid profiles [33–35]. For example, a prudent diet consisting of a high intake of fruits, vegetables, seafood, whole cereal and low-fat dairy products was associated with reduced plasma TC (− 12.0 mg/dL), LDL-C (− 12.0 mg/dL), and apo B (− 6.6 mg/L) among women in Latin America [33]. Also, adherence to a Mediterranean dietary pattern increased the HDL-C levels and decreased the TG/HDL-C ratio when compared to a Western dietary pattern [34]. Similarly, healthy Korean diet (which is high in whole grains, legumes, nuts, vegetables, mushrooms, and fruits) improved the lipid profiles among Korean adults with type 2 diabetes [35].

Immigrants in the US tend to adopt a more Westernized dietary pattern, a major contributor of growing rate of obesity, one of the prominent risk factors of CVD, in the US from 30.5% (1999–2000) to 41.9% (2017–2020) [36]. In the current study, Filipino immigrant women in Korea who were exposed to a Korean diet and dietary practices had a lower cholesterol and TG level but had a higher mean HDL-C (58.19 ± 14.06 mg/dL) compared to the FW in the Philippines (HDL-C 40.26 ± 11.80 mg/dL). KW were exposed to a more varied diet compared to FW in the Philippines [31, 37]. The Korean diet traditionally consists of cooked rice, dishes with broth, and small dishes (side dishes mostly seasoned or fermented), with a proportionally high vegetable intake, moderate to high legumes and fish intake, and low red meat intake [38]. Individuals from different dietary traditions showed relatively stable dietary habits despite the occasional deviation, and accordingly, the prognosis of CVD risk factors might differ among communities, families, and socioeconomic strata [11]. Studies have shown that exposure to a host culture may lead to changes in diet and health outcomes [27]. However, the molecular and cellular mechanisms by which dietary components affect CVD risk factors may be complex and yet to be clearly understood [39]. Further prospective studies are needed to evaluate the dietary mechanisms. We can also attribute this unexpectedly better lipid profiles of FW in Korea compared to FW in the Philippines to the healthy immigrant effect [40]. In this current study, this theory might implies that FW in Korea are exposed to varied and healthier Korean diet regardless of their socio-economic status after they came. Although it is not difficult to adjust to different food cultures no matter how beneficial, FW in Korea might find it challenging. Although this healthy immigrant effect alone could not explain the reason for these disparities between immigrants and non-immigrants Filipino, it could at least broaden our understanding of the susceptibility and prognosis of CVD risk factors among FW in Korea. However, this theory need further study to warrant this account.

The reasons why we observed unfavorable lipid profiles among FW in the Philippines compared to Korean are not clear but warrants further studies. It is possible that the transition to unhealthy lifestyle factors may be associated with unfavorable lipid profiles among Filipino women [11]. In the Philippines, the prevalence of elevated TC increased from 28.0% (2003) to 47.2% (2013). Similarly, the proportion of Filipinos with an elevated LDL-C profile increased by more than 15% between 2003 and 2013 from 31.5% (2003) to 47.5% (2013) and for low HDL-C from 54.2% (2003) to 71.0% (2013), particularly among women [41].

We noticed that FW in Korea presented a higher prevalence of hypertension when compared to KW. A change in the food environment after migration might gradually change their dietary habit. Our study showed that FW in the Philippines had the lowest mean percent energy from fat, but after migrating, the mean percent energy from fat of FW in Korea was higher than KW. Koreans have one of the highest salt intake worldwide, almost half of which is derived from traditional diets such as salted vegetable (*kimchi*) [42]. Excessive sodium consumption significantly increased BP and CVD complication [43]. Exposure to an unhealthy diet, including the Korean diet high in sodium and the traditional Filipino diet high in fat (e.g., deep-fried foods), could presumably be the culprit of the overall high prevalence of hypertension among FW in Korea. In addition to dietary factors, genetic factors may be related to an increase in BP. Several genetic variants predict the risk of developing hypertension among normotensive individuals in GenSalt study [44]. Although there have been studies examining the genetic variants associated with hypertension among East Asians [45], but such studies are scarce among Filipinos, and it is difficult to elucidate the complexity of genetics affecting hypertension among FW in Korea. Obesity is a risk factor for hypertension. However, adjustment for obesity appreciably attenuated the positive association among FW in the Philippines and in Korea, suggesting the mediation effect by obesity. The reason why we found a higher prevalence of obesity warrants further studies, including how socioeconomic segregation influences the susceptibility of women to obesity [46].

Unhealthy dietary habits and the lifestyle of Filipinos may be important in the significant transitions of CVD events in the last decade. A recent review reported several risk factors of CVD prevalent among Filipino immigrants in the US. The study had indicated that Filipinos tend to develop an acculturated Westernized diet, which is associated with a higher risk for CVDs, hypertension, type 2 diabetes, and metabolic syndrome [14]. The prevalence of CVD risk factors among Filipino immigrants in the US might due to higher rates of smoking (17.7%) [12], lower levels of HDL-C (mean ± SD: 40.8 ± 0.2 mg/dL) [47], overweight or obesity (BMI ≥ 25 kg/m^2^, 60.4%) [48], abdominal obesity (> 88 cm, 84.9%) and physical inactivity (48%) [49], high carbohydrate, sodium and fat diet [50, 51], and chronic conditions such as dyslipidemia (30.2%), diabetes (8.7%) and hypertension (41.2%) which are known CVD risk factors [52]. Additionally, the prevalence of CVD risk factors among Filipino American women increased at a BMI as low as 23–24.9 kg/m^2^ to ≥ 30 kg/m^2^, including diabetes (HbA1c ≥ 6.5%, 6.38 ± 2.61 to 6.40 ± 1.08, respectively) and hypertension (SBP ≥ 140 mmHg, 125 ± 15 to 139 ± 19, respectively; or DBP ≥ 90 mmHg, 81 ± 8.4 to 87 ± 12, respectively), at a higher TG (≥ 150 mg/dL, 110 ± 55 to 142 ± 79, respectively), and at a lower HDL-C level (≤ 60 mg/dL, 60 ± 13 to 55 ± 14, respectively) [53].

To the best of our knowledge, our study is the first to compare the susceptibility of CVD risk factors among Filipino and Korean women of diverse backgrounds matched by age (1:1 ratio). Studies conducted on Filipino immigrants in South Korea are sparse and out-of-date. Thus, the literature is limited to support the account regarding the Filipino immigrants’ health status. Another strength of our study is the multivariate adjustment for potential confounders. Some limitations of our study warrant consideration in the interpretation of the results. First, the cross-sectional design of our study delimits the inference of causality. Second, the FiLWHEL study has a relatively small sample size based on convenience sampling which may not represent the general population. Third, we did not adjust for physical activity even though it is a known risk factor of CVD because of the different questionnaires used in collecting physical activity information across the studies. However, adjustment of BMI or waist circumference may take into account the adjustment for physical activity levels to some degree.

## Conclusions

The prevalences of high TC, low HDL-C, high LDL-C and high TG were higher among FW in the Philippines than FW in Korea and KW. FW in Korea had higher prevalences of obesity and hypertension than KW. Still, there was a similarity between the lipid profiles of FW in Korea and those of KW.

## Supplementary Information

**Additional file 1. Fig. S1**: Flow chart of age-matched population of FiLWHEL, NNS, and KNHANES participants.

## Data Availability

The authors declare that all supporting files of the findings of this study can be found within the article. In addition, the datasets used during the analysis of the current study can be acquired from the corresponding author on reasonable request or upon specified agreement.

## Abbreviations

BMI: Body mass index
BP: Blood pressure
CDC: Centre for Disease Control
CV: Coefficient of variation
CVD: Cardiovascular disease
FiLWHEL: Filipino Women's Health and Diet Study
FNRI: Food and Nutrition Research Institute
FW: Filipino women
HDL-C: High-density lipoprotein cholesterol
IASO/IOTF: International Association for the Study of Obesity/International Obesity Task Force
IDF: International Diabetes Federation
KDCA: Korea Disease Control and Prevention Agency
KNHANES: Korean National Health and Nutrition Examination Survey
KW: Korean women
LDL-C: Low-density lipoprotein cholesterol
MEC: Mobile Examination Center
NCEP: National Cholesterol Education Program
NHANES: National Health and Nutrition Examination Survey
NHLBI: National Heart, Lung, and Blood Institute
NNS: National Nutrition Survey
TC: Total cholesterol
TG: Triglyceride
US: United States
WHO: World Health Organization
