# Supplementary material for: Comparison of cardiovascular disease risk factors among FiLWHEL (2014–2016), NNS (2013) and KNHANES (2013–2015) women
Source: BMC Womens Health. 2023 Mar 30;23:149. doi: 10.1186/s12905-023-02218-1 (PMC10064574; doi:10.1186/s12905-023-02218-1)
Supplement: Supplementary file 1 — Additional file 1. Fig. S1: Flow chart of age-matched population of FiLWHEL, NNS, and KNHANES participants. [file 12905_2023_2218_MOESM1_ESM.docx]

Total FiLWHEL participants

**(n=504),** all available for matching

**Matching Criteria**

∙ Age: 20-57 years old

∙ Sex: Female

∙ Marital Status: married for NNS only

∙ Biochemical data: TC, TG, HDL-C, LDL-C, and glucose

**Supplemental Figure 1.** Flow chart of age-matched population of FiLWHEL, NNS, and KNHANES participants.

**n=5044**

**n=5044**

Available for random sampling **(n=1,739)**

Available for random sampling **(n=4,646)**

**Excluded Variables**

∙ Male (n=10,411)

∙ Aged <20 or >57 years old (6,257)

∙ No blood lipids or glucose (n=4,541)

**Excluded Variables**

∙ Male (n=87,083)

∙ Aged <20 or >57 years old (n=46,876)

∙ Not married (n=16,210)

∙ No blood lipids or glucose (n=17,519)

Total NNS participants

**(n=172,334)**

Total KNHANES participants **(n=22,948)**

(n=
